# Emergency Mechanical Thrombectomy for Acute Middle Cerebral Artery Occlusion Accompanied by Adult-Onset Still’s Disease

**DOI:** 10.7759/cureus.59196

**Published:** 2024-04-28

**Authors:** Junpei Nagasawa, Makiko Ogawa, Hiromi Konaka, Masaru Yanagihashi, Osamu Kano

**Affiliations:** 1 Neurology, Toho University Faculty of Medicine, Tokyo, JPN

**Keywords:** embolic cerebral infarction, mechanical thrombectomy (mt), large vessel occlusion (lvo), adult-onset still’s disease, acute cerebral infarction

## Abstract

Adult-onset Still’s disease (AOSD) is a rare systemic inflammatory condition of an unknown etiology. Stroke is a rare complication associated with AOSD; most of these are cerebral infarctions due to the occlusion of small blood vessels. Here, we report the first case of mechanical thrombectomy in a patient with cerebral infarction due to a large vessel occlusion associated with AOSD.

A 60-year-old man with no underlying disease was diagnosed with AOSD. Sixteen days after admission, he suddenly lost consciousness and was found to have right hemiplegia and aphasia. Head CT showed early signs of ischemic infarction in the left insular cortex, and head CT angiography demonstrated occlusion in a part of the left middle cerebral artery (MCA). Therefore, we decided that mechanical thrombectomy was an indication of revascularization. We performed mechanical thrombectomy using a Trevo NXT 4 × 28 mm (Stryker, Kalamazoo, USA) and obtained reperfusion of the MCA. The results of the cerebral angiography were indicative of an embolic cerebral infarction, and we investigated the source of the embolism including an insertable cardiac monitor (ICM) (Reveal LINQ, Medtronic, Minneapolis, USA). However, no disease other than AOSD that could be a source of embolism was observed. Therefore, AOSD was assumed to be associated with embolisms.

AOSD may cause embolic cerebral infarction and may be indicated for mechanical thrombectomy.

## Introduction

Adult-onset Still’s disease (AOSD) is a rare systemic inflammatory condition of an unknown etiology. Stroke is a rare complication associated with AOSD [1], and most of these are cerebral infarctions due to the occlusion of small blood vessels, associated with vasculitis or thrombotic microangiopathy. Here, we report a case of mechanical thrombectomy in a patient with cerebral infarction due to middle cerebral artery occlusion associated with AOSD. Two weeks after being diagnosed with AOSD, he developed a severe cerebral infarction, and his symptoms improved with a mechanical thrombectomy. We diagnosed the type of cerebral infarction as embolism. Therefore, screening tests for embolic sources, including 24-hour Holter electrocardiographic monitoring and insertable cardiac monitor (ICM), were performed but no causes besides AOSD were found. He was treated with aspirin and showed no recurrence of cerebral infarction. To the best of our knowledge, there are no previous reports of patients with AOSD having large vessel occlusion and undergoing mechanical thrombectomy.

## Case presentation

A 60-year-old man with no underlying disease was admitted to the Department of Collagen Disease in our hospital. He had a fever, joint pain, and a rash on his thighs that had lasted for two months. After examination, he was diagnosed with AOSD based on Yamaguchi's criteria [2] and treated with prednisolone (PSL) 60 mg daily and methotrexate (MTX) 8 mg weekly. 

**Figure 1:**
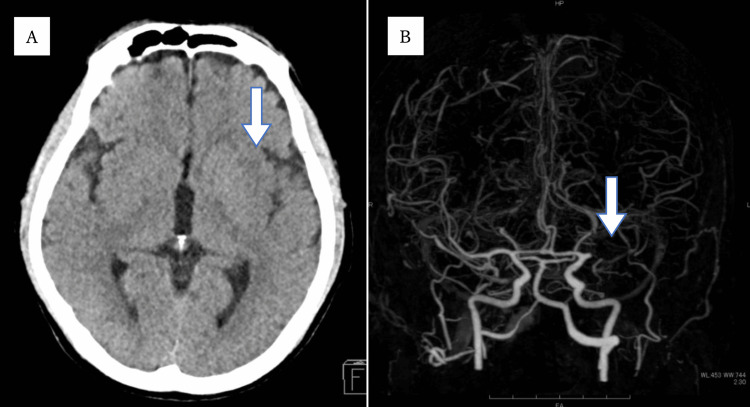
Initial CT scan and CT angiography Head CT showed early signs of ischemic infarction in the left insular cortex (A: arrow). Head CT angiography indicated occlusion in the horizontal part of the left middle cerebral artery (B: arrow).

Sixteen days after admission, he was found unconscious by a visiting nurse. The time of onset was unknown, but five hours had already passed since last known well. The attending physician urgently consulted with us in the Department of Neurology. On examination, right hemiplegia, left conjugate deviation, and aphasia were noted. The National Institute of Health Stroke Scale (NIHSS) score was 20. A stroke was suspected based on the findings, and imaging tests were conducted. Head CT showed early signs of ischemic infarction in the left insular cortex, and head CT angiography demonstrated occlusion in a part of the left middle cerebral artery (MCA) (Figure 1). The time from the last known well to CT scan was 330 minutes. It was difficult to perform an emergency magnetic resonance imaging (MRI) at our hospital. By the time the medical examination was performed, five hours had already passed since the last known well. Therefore, intravenous thrombolysis was not indicated, and we decided that mechanical thrombectomy was an indication for revascularization.

**Figure 2:**
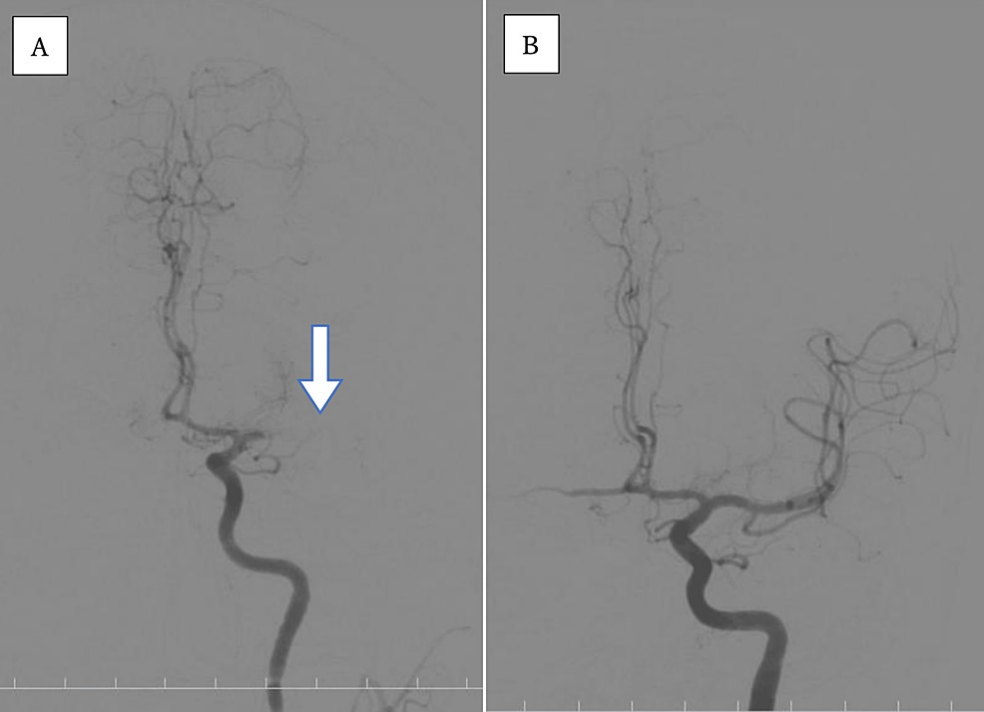
Emergency mechanical thrombectomy Angiography of the left internal carotid artery showed occlusion of the proximal M1 segment of MCA (A: arrow). After a Trevo NXT 4 × 28 mm (Stryker, Kalamazoo, USA) was deployed at the obstructed part of the left MCA, the angiography showed reperfusion of MCA (B). MCA: Middle cerebral artery

**Figure 3:**
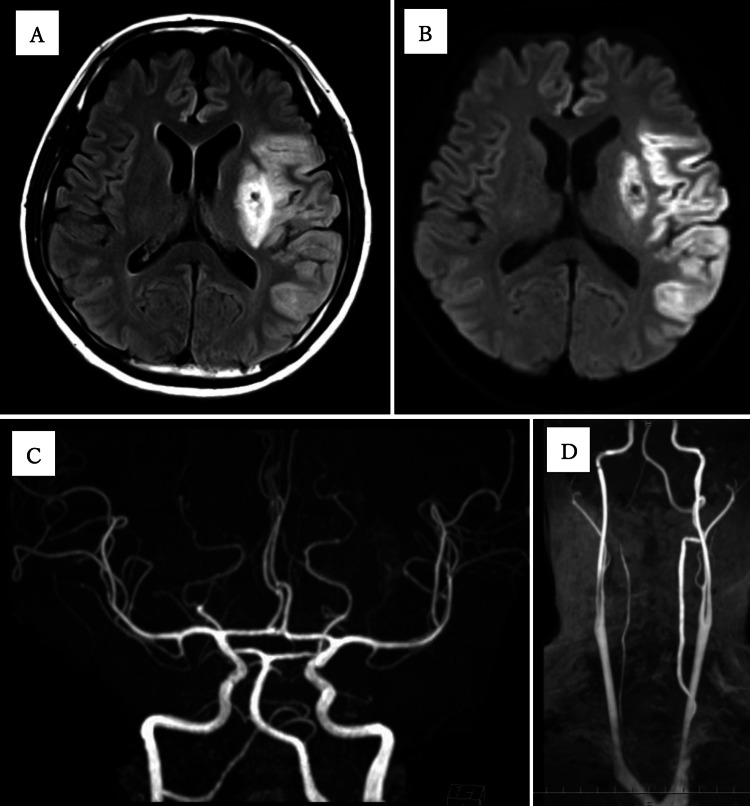
Brain MRI performed two days after onset Fluid-attenuated inversion recovery (A) and diffusion-weighted imaging (B) revealed an infarct in the left MCA region. Brain MR angiography (C) and neck MR angiography (D) revealed no stenotic lesions or obvious plaque lesions. MCA: Middle cerebral artery; MR: Magnetic resonance

Angiography of the left internal carotid artery revealed occlusion of the proximal M1 segment of MCA (Figure 2A). A Trevo NXT 4 × 28 mm (Stryker, Kalamazoo, USA) was deployed at the obstructed part of the left MCA through a Trevo Track 21 microcatheter (Stryker, Kalamazoo, USA). Immediately after angiography, reperfusion of the MCA (Figure 2B) and thrombolysis in cerebral infarction (TICI) grade was 2b. The time from last known well to puncture was 380 minutes, and the time from puncture to recanalization was 45 minutes. The following day, his neurological findings improved, with an NIHSS score of 12. MRI performed two days later confirmed an infarct in the left MCA region and a recanalized left MCA, and there were no stenotic lesions suggestive of vasculitis (Figure 3). 

The results of the cerebral angiography were indicative of an embolic cerebral infarction, and the source of the embolism was not identified. Furthermore, transthoracic echocardiography, transesophageal echocardiography, continuous electrocardiogram monitoring for seven days after admission, and 24-hour Holter electrocardiographic monitoring revealed no cardioembolic risk factors such as paroxysmal atrial fibrillation (Paf), left atrial thrombus, infective endocarditis, patent foramen ovale (PFO), or heart valve disease. CT angiography, magnetic resonance (MR) angiography (Figures 3C-3D), and vascular ultrasonography revealed no plaque lesions that could be a source of artery-to-artery embolism. Pathological examination revealed a thrombus, rich in fibrin and red blood cells.

A laboratory test showed the following results: white blood cells: 20.2 × 10^3^/μL, neutrophils: 16.5 × 10^3^/μL, C-reactive protein: 7.5 mg/dL, erythrocyte sedimentation rate: >100 mm, hemoglobin: 12.6 mg/dL, platelets: 383 × 10^3^/μL, aspartate transaminase (AST): 86 IU/L, alanine transaminase (ALT): 122 IU/L, prothrombin time (PT)-international normalized ratio (INR): 1.1 (n.v. < 1.2), activated partial thromboplastin time (aPTT): 24.5 sec, and D-dimer: 1.0 μg/mL. 

Workup for autoimmune disorders performed were as follows: lupus anticoagulant was performed using diluted Russell viper venom time test and was negative. Anticardiolipin (aCL) antibody IgG, aCL antibody IgM, and antineutrophil cytoplasmic antibody were also negative. Antinuclear antibody was negative as well; anti-dsDNA antibody testing was not conducted.

From these results, blood sampling tests did not reveal any diseases that could cause coagulation abnormalities, such as antiphospholipid antibody syndrome, anticoagulant factor deficiency, or blood platelet disorder. Based on these results, the patient was diagnosed with embolic stroke of an undetermined source. Consequently, an ICM (Reveal LINQ, Medtronic, Minneapolis, USA) was implanted to find Paf. On the 60^th^ day after admission, the patient was transferred to a rehabilitation hospital with a modified Rankin Scale (mRS) score of 4. Currently, approximately one year after the onset of cerebral infarction, Paf has not been detected by ICM. Therefore, it is difficult to assume that Paf was the cause of the cerebral infarction. Therefore, AOSD was assumed to be associated with embolisms. He has been treated with aspirin and showed no recurrence of cerebral infarction, and his follow-up mRS score was 2. Treatment with PSL and MTX was continued, and the patient’s fever, skin rash, and joint pain improved. PSL, which was started at 60 mg, was gradually reduced and controlled at 5 mg or less. MTX is currently being continued at 10 mg.

## Discussion

To the best of our knowledge, this is the first report of mechanical thrombectomy for cerebral infarction associated with large-vessel occlusion in a patient with AOSD.

The association between AOSD and vasculitis of small blood vessels has been reported based on the pathological findings of a salmon-pink rash and elevated markers for vasculitis in AOSD [3]. Previously, a patient with multiple cerebral infarctions was diagnosed with AOSD, and the cerebral infarctions were assumed to be related to vasculitis [4]. 

Association has also been reported between AOSD and thrombotic microangiopathy. In more than half the patients, thrombotic microangiopathy occurred within the first six months after the diagnosis of AOSD [5]. As described above, thrombocytosis or vasculitis is a major cause of cerebral infarction in AOSD patients. However, its exact mechanism of action remains unknown. 

This case involved an embolic cerebral infarction, and a thrombus was likely formed in the artery or heart. While we conducted several tests, none showed any source of embolism, and ICM confirmed the absence of Paf. Therefore, AOSD may be associated with blood clots and cerebral infarction. Furthermore, venous thrombosis is likely to occur with AOSD [6], but in this case, there was no deep vein thrombosis on lower limb ultrasonography or right-to-left shunt disease, such as PFO; therefore, venous thrombosis was not considered to be the cause of cerebral infarction.

There are few reports of cerebral infarction due to large-vessel occlusion in patients with AOSD. Other than this case, there is only one study by Goh et al., reporting a patient with AOSD having an acute ischemic stroke secondary to a floating internal carotid artery thrombus. They postulated that hyperviscosity from thrombocytosis, hypercoagulability from the underlying inflammatory state, and vasculitis might all contribute to acute ischemic stroke [7]. 

We suggest that AOSD may be involved not only in small blood vessel occlusion but also in large vessel occlusion due to thrombus embolism, considering this case and Goh’s case. Because AOSD is a rare disease and there is little knowledge about its relationship with cerebral infarction, it is necessary to accumulate more cases in the future.

## Conclusions

AOSD may cause embolic cerebral infarction and may be indicated for mechanical thrombectomy. Collagen diseases, including AOSD, can promote blood clot formation or cause vasculitis, which can lead to cerebral infarction, so care must be taken to avoid delays in diagnosis.
